# Mapping the STK4/Hippo signaling network in prostate cancer cell

**DOI:** 10.1371/journal.pone.0184590

**Published:** 2017-09-07

**Authors:** Damien Ready, Kader Yagiz, Pooneh Amin, Yuksel Yildiz, Vincent Funari, Serdar Bozdag, Bekir Cinar

**Affiliations:** 1 Department of Mathematics, Statistics, and Computer Science, Marquette University, Milwaukee, Wisconsin, United States of America; 2 Department of Medicine, Samuel Oschin Comprehensive Cancer Institute, Cedars-Sinai Medical Center, Los Angeles, California, United States of America; 3 Department of Biological Sciences, the Center for Cancer Research and Therapeutic Development, Clark Atlanta University, Atlanta, Georgia, United States of America; 4 Department of Physiology, Adnan Menderes University, Aydin, Turkey; 5 Department of Medicine and Division of Genetics, Cedars-Sinai Medical Center, Los Angeles, California, United States of America; University of British Columbia, CANADA

## Abstract

Dysregulation of MST1/STK4, a key kinase component of the Hippo-YAP pathway, is linked to the etiology of many cancers with poor prognosis. However, how STK4 restricts the emergence of aggressive cancer remains elusive. Here, we investigated the effects of STK4, primarily localized in the cytoplasm, lipid raft, and nucleus, on cell growth and gene expression in aggressive prostate cancer. We demonstrated that lipid raft and nuclear STK4 had superior suppressive effects on cell growth *in vitro* and *in vivo* compared with cytoplasmic STK4. Using RNA sequencing and bioinformatics analysis, we identified several differentially expressed (DE) genes that responded to ectopic STK4 in all three subcellular compartments. We noted that the number of DE genes observed in lipid raft and nuclear STK4 cells were much greater than cytoplasmic STK4. Our functional annotation clustering showed that these DE genes were commonly associated with oncogenic pathways such as AR, PI3K/AKT, BMP/SMAD, GPCR, WNT, and RAS as well as unique pathways such as JAK/STAT, which emerged only in nuclear STK4 cells. These findings indicate that MST1/STK4/Hippo signaling restricts aggressive tumor cell growth by intersecting with multiple molecular pathways, suggesting that targeting of the STK4/Hippo pathway may have important therapeutic implications for cancer.

## Introduction

Mammalian STE20-like serine-threonine kinase MST1, encoded by the STK4 gene, is a multifunctional protein [1, 2]. MST1 and its closest paralogs MST2 (encoded by the STK3 gene), MST3, and MST4 are members of the Class II Germinal Center Family of Protein Kinases [3]. Here, we use STK4, an official gene name for MST1, to avoid confusion with the MST1 official gene name that encodes macrophage stimulating 1 or hepatocyte growth factor-like protein. STK3/4 and LATS1/2 (large tumor suppressor 1 and 2) are core kinase components of the Hippo tumor suppressor pathway in mammalians [4]. In the conventional Hippo pathway, the STK3/4 and LATS1/2 signaling cascade phosphorylates and inactivates the transcriptional coactivator YAP1 (yes associated protein 1) and its close paralog WWTR1 [5]. YAP1 and WWTR1 do not have DNA binding domains and they exert their biological outputs, such as cell proliferation and survival, by interacting with the TEAD1-4 transcription factors.

Lines of evidence have indicated that dysregulation or loss of STK4/Hippo signaling is linked to developmental disorders and carcinogenesis with poor prognosis [6–12]. For example, a genetic deletion of hippo (*hpo*) in *Drosophila* results in a tumor-like phenotype due to the loss of apoptosis [13]. Similarly, mice with the conditional STK3/4 gene knockouts show stem cell expansion, and tumorigenesis [11, 14, 15]. STK4 is a stress-induced kinase and it can be activated in response to cell-death inducers. Autophosphorylation of STK4 at Thr183 (Thr180 in STK3) in the activation loop is a key activation mechanism for STK4/3 because phosphorylation of Thr183/180 causes the cleavage of STK4 by caspases under apoptotic conditions [3, 16, 17]. The caspase-cleavage results in a more active STK4 protein (STK4-N, an amino-terminally truncated STK4), which localizes into the nucleus and induces apoptosis through histone modifications and chromatin condensations [18, 19].

Previously, we identified STK4 as a binding partner of AKT protein complexes that were isolated from lipid raft of the androgen-sensitive LNCaP prostate cancer (PC) cell line [7]. Lipid raft is the specialized cholesterol-rich membrane microdomain and plays a critical role in signal transductions and cell survival [20–23]. In that study, we demonstrated that levels of STK4 protein progressively declined during PC progression to the metastatic castration-resistant state, which coincided with the activation of AKT1 [7, 24]. In addition, we and others reported that DNA hypermethylation [17, 25] and post-translational modification [17, 25] meadiated the loss of STK4 activity. Interestingly, a recent study suggested that the dimerization of STK3 and STK4 that was mediated by H-ras signaling caused the loss of STK4 activity [26]. Moreover, we reported that the full-length STK4 (STK4-FL) enriched in cell nuclei, even in the presence of cell-death inducer, was devoid of Thr183 phosphorylation [25]. Nevertheless, how STK4 in a defined cell location regulates PC cell growth remains elusive.

In the present study, we developed and utilized the cytoplasm-, lipid raft- and nuclear-localized STK4 expressing PC cell models to gain more insights into the role of STK4 in aggressive PC. We found that STK4 enriched in the defined subcellular compartment differentially regulated cell growth *in vitro* and tumor growth *in vivo*. We identified several differentially expressed (DE) genes that responded to the enrichment of ectopic STK4 in all three cell compartments. Our functional annotation clustering showed that these DE genes were associated with a wide range of molecular pathways including tumor suppressor and oncogenesis as well as cellular metabolisms. Our findings suggest that STK4 signaling controls aggressive prostate tumor cell growth by modulating with multiple signaling mechanisms.

## Materials and methods

### Plasmids

Construction of the tetracycline (Tet) or doxycycline (Dox)-inducible STK4 plasmid (pRX-HA-STK4) was described previously [27]. Dox is a tetracycline analog. To express Hemagglutinin (HA)-tagged STK4 protein in the lipid raft membrane domain, we constructed lipid raft (LR)-targeted pRX-LR-HA-STK4 mammalian expression vector. To generate pRX-LR-HA-STK4 vector, we took a series of approaches. First, we generated a pRX-LR-HA vector, for which double-stranded 5’-phosphorylated DNA consisting of palmitoylation and myristoylation (PM) signal from the Lck gene and the HA tag sequences were ligated into the BamH1 and NotI restriction enzyme (RE) sites in the pRetro-X-Pur (Pur: puromycin) retroviral vector (Clontech Laboratories, Inc.). The resulting vector was designated as pRX-PM-HA. Second, the PCR-amplified full-length STK4 cDNA was inserted into the NotI and MluI enzyme sites in the pRX-PM-HA vector. To express HA-tagged STK4 protein in the nucleus, we constructed nuclear (NL)-targeted pRX-NL-HA-STK4 mammalian expression vector. To construct pRX-NL-HA-STK4, first, we generated pRX-3NLS-HA vector, for which double-stranded 5’-phosphorylated DNA containing three consecutive copies of nuclear localization signal (3NLS) from SV40 large T-antigen separated with three base-pair spacers and HA-tag sequences were ligated into the BamH1 and NotI RE sites in the pRetro-X-Pur retroviral vector. The resulting vector was designated as pRX-NLS-HA. Second, the PCR-amplified full length STK4 cDNA was inserted into the NotI and MluI RE sites in the pRX-3NLS-HA vector. AccuPrime^™^ Pfx SuperMix (Invitrogen; Grand Island, NY) was used in PCR reactions. Standard molecular biology techniques in cloning and DH5-α competent cells in plasmid amplification were utilized [27]. In-frame and fidelity of all constructs were confirmed by DNA sequencing.

### Cell models

**Fig 1:**
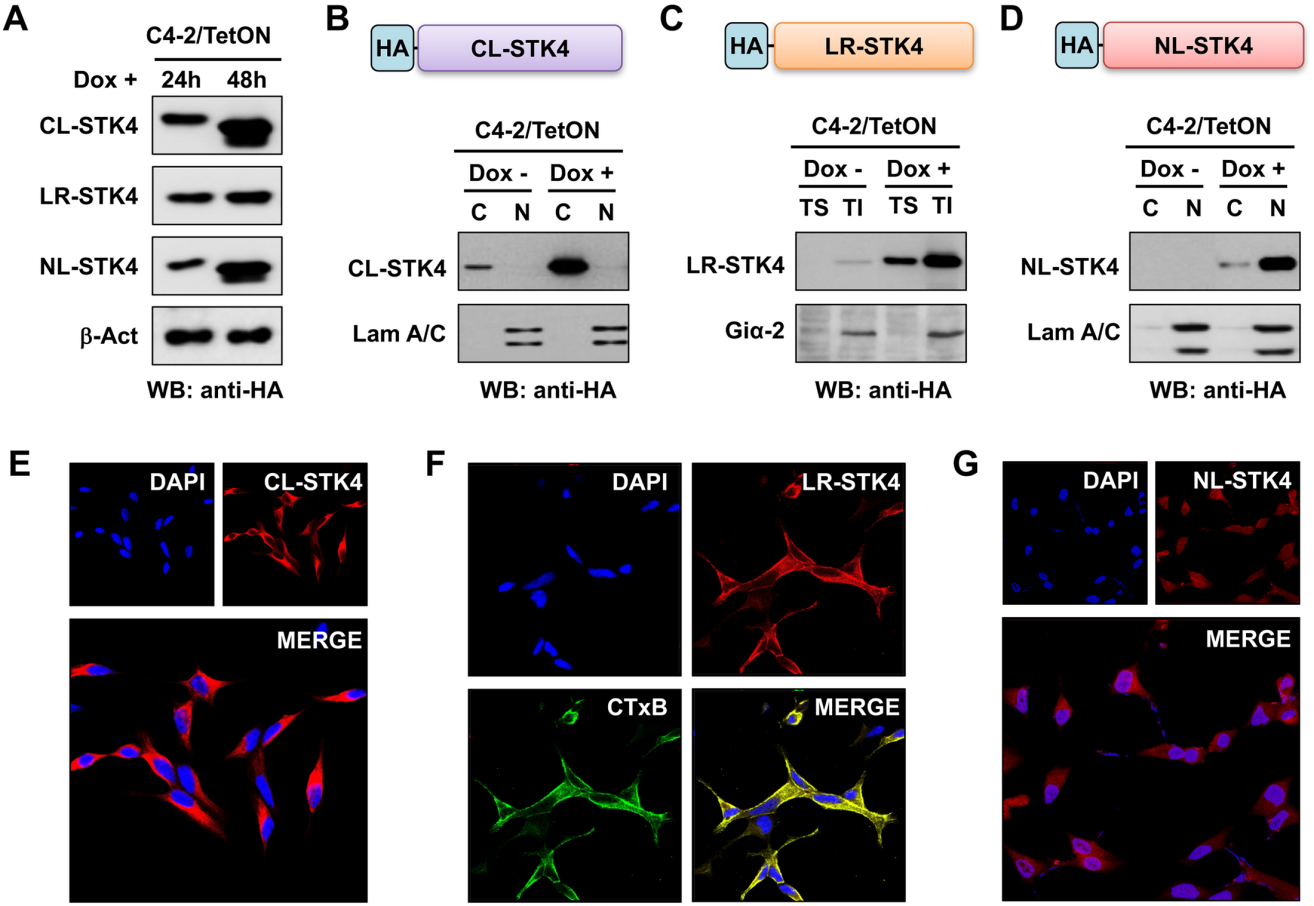
Expression of CL-STK4, LR-STK4, and NL-STK4 protein in Tet-inducible C4-2 prostate cancer cells. **(A)** Time-dependent expression of CL-STK4, LR-STK4, and NL-STK4 protein in the engineered cells that were exposed to doxycycline (Dox) for 24 and 48h. Levels of ectopic STK4 proteins were assessed by Western blotting (WB) using the HA-tagged antibody. (**B-D)** Analysis of CL-STK4, LR-STK4, and NL-STK4 protein in cytoplasmic, lipid raft, and nuclear fractions, respectively. Expression of HA-STK4 protein was evaluated by WB with the HA-tag antibody at 48h after treatment with and without doxycycline (Dox, 4 μg/ml). Lam (lamin) A/C was used as a nuclear marker. G_i_α2 was used as a lipid raft marker. **(E-G)** Immunofluorescence (IF) analysis of CL-STK4, LR-STK4, and NL-STK4 protein in the C4-2/CL-STK4, C4-2/LR-STK4, and C4-2/NL-STK4 cells, respectively. IF was performed at 24h post Dox (4 μg/ml) treatment. Micrographs are the representation of two independent experiments. CTxB-FITC labeled lipid raft in C4-2/LR-STK4 cells. For both experiments, cells were grown in Tet-approved serum conditions. CL: Cytoplasmic localization, LR: Lipid raft; NL: nuclear localization, HA: Hemagglutinin.

Establishment of Tet or Dox-responsive C4-2/Vector and C4-2/HA-STK4 cell models was previously described [27]. Here, we renamed the C4-2/HA-STK4 cell as C4-2/CL-STK4 because we noted that ectopic expression of HA-STK4 was naturally accumulated in the cytoplasm ([27] and Fig 1A and 1D). To establish the C4-2/LR-STK4 cell model, first retrovirus carrying pRX-LR-HA-STK4 and pRX-NL-HA-STK4 vector were produced in HEK-293T cells as previously described [27]. Then, C4-2/TetON cells were infected with retrovirus encoding pRX-LR-HA-STK4 or pRX-NL-HA-STK4 vector, followed by Puromycin (Pur) selection (3 μg/mL) to generate Tet-inducible C4-2/LR-HA-STK4 or C4-2/NS-HA-STK4 expressing cells. We designated these cells as C4-2/LR-STK4 and C4-2/NL-STK4, respectively. All protocols and procedures were performed according to the manufacturer's instructions (Clontech Laboratories, Inc.). Growth conditions for C4-2 and HEK 292T cells were previously described [27].

### Protein analysis

Cytoplasmic and nuclear fractions were prepared using our established method [28]. TS (Triton-X-100-soluble) fraction and TI (Triton-X-100 insoluble, but *n*-octyl-β-D-glucoside soluble) fraction—TI fraction by definition, represents lipid raft—were isolated according to the established protocol [7, 29]. Presence of ectopic HA-tagged STK4 protein in cytoplasmic, lipid raft and nuclear fractions was determined by Western blotting using the HA antibody (Covance). Briefly, proteins were resolved by SDS-PAGE. PBST (0.1% Tween-20) containing 5% (w/v) skim milk powder or PBST containing 5% immunoglobulin G (IgG)-free bovine serum albumin (Sigma-Aldrich) was used in membrane blocking and antibody dilutions. Giα2 protein was included as a negative and positive control for TS and TI fractionations, respectively. Lamin A/C (Cell Signaling Technology) was used as a negative and positive control for cytoplasmic and nuclear fractionations, respectively [27]. Signals were visualized by chemiluminescence method (GE HealthCare).

### Growth assays

C4-2/Vector, C4-2/CL-STK4, C4-2/LR-STK4 and C4-2/NL-STK4 cells were seeded in tissue culture medium supplemented with 10% Tet-approved serum in 96-well cell culture plate. Cells were then treated with Dox (3 μ/ml) for 72h to induce STK4 expression in the cell. Cell growth was assessed by imaging and MTS assay. For imaging, cells were washed with PBS and bright-field images were captured using microscopy (Nikon Eclipse *Ti* model; USA) at 20x magnification. CellTiter 96 AQueous system was used to assess cell growth according to manufacturer’s protocol (Promega) and as described [27]. This system uses MTS and it has been widely used to evaluate cell growth in cultures [27].

### Immunofluorescence

Immunofluorescence analysis of HA-STK4 protein in C4-2 cells was performed with modifications [27]. Briefly, cells were fixed with freshly prepared 4% PFA (paraformaldehyde that was prepared in PBS) for 30 min and permeabilized with 0.2% Triton-X-100 and incubated with anti-HA (Covance, 1:50) antibody overnight at 4°C. Cells were washed with PBS after each step. In addition, lipid rafts were labeled with CTxB-FITC conjugated (Sigma-Aldrich) as described [21]. Briefly, live cells were washed with cold PBS and incubated with CTxB-FITC (20 ng/ml, which was prepared in cold serum-free media) on ice for 30 min prior to fixation with 4% PFA. Alexa Fluor 532 conjugated anti-mouse (1:1000 dilution) was used to detect HA-STK4 signals in the cell. Slides were mounted with VectaShield containing DAPI (Vector Labs, H-1200). Immunofluorescence images were captured by microscopy (Zeiss 700) at 40x magnification with oil immersion.

### Xenograft assays

C4-2/Vector, C4-2/LR-STK4, and C4-2/NL-STK4 cells mixed with Matrigel (1:1 ratio) were implanted subcutaneously at the right and left flanks of the nude and immunocompromised male mice (n = 10 per condition). 1×10^6^ cells/100 μL were used per injection per site under anesthesia by isoflurane. Starting 24h post cell inoculation, mice were treated with Dox (0.5 mg/mL) in drinking water for 6 weeks to induce STK4 expression. A weekly tumor size measurement was assessed by caliper manually [27, 29]. Upon completion of the experiment, mice were sacrificed by humane way (by CO_2_ inhalation, followed by cervical dislocation) and tumor tissues extracted from mice were fixed in 5% formaldehyde or “snap” frozen at −80°C for future analysis. Animal study was conducted in strict accordance with the recommendations in the Guide for the Care and Use of Laboratory Animals of the National Institutes of Health. The animal protocol was approved by the Institutional Animal Care and Use Committee (IACUC) at Cedars-Sinai Medical Center. All experimental procedures in live animals were performed under isoflurane anesthesia and all efforts were made to minimize suffering. Mice were euthanized by CO_2_ inhalation, followed by cervical dislocation.” Student's t-test (two-tailed) was used to determine the significance between the two groups. *P*-value ≤ 0.05 was considered significant.

### RNA isolation and sequencing

Total RNA in biological replicates was isolated from C4-2/Vector, C4-2/CL-STK4, C4-2/LR-STK4, and C4-2/NL-STK4 cells using RNA isolation kit according to manufacturer’s instruction (Life Technologies). Cells were grown in 10% Tet-approved serum-fed conditions at 80% confluence prior to RNA isolation. Quality of total RNA was assessed prior to library construction. RNA-sequencing (RNAseq) libraries were prepared using the standard protocol and sequenced by Illumina Genome Analyzer IIx in Genomic Core at Cedar-Sinai Medical Center.

### RNAseq data analysis

The quality of 72 base-pair reads was assessed with FastQC 0.10.1 software [30]. FastQC identifies lingering TruSeq adapter sequences present on reads. We used cutadapt tool to trim low quality base pairs and the TruSeq adapter sequences from the end of reads [31]. The reads were then aligned against the Illumina iGenomes Homo Sapiens NCBI build 37.2 reference sequence using Tophat 2.2.08b [32]. To compute DE genes, gene counts (i.e., number of sequence reads assigned to each gene) were calculated using htseq-count tool [33]. The gene count data were fed into DESeq2 Bioconductor package in R to identify DE genes using the false discovery rate (FDR) and fold change cutoffs [34]. To demonstrate the consistency of transcript abundance between replicates, scatterplot of the DESeq2 normalized gene counts were plotted in R.

### Gene ontology and pathway enrichment analysis

To compute the functional annotation of the DE genes, we performed gene ontology (GO) [35] and KEGG pathway [36] enrichment analysis using STRINGDb Bioconductor package (version 1.14.0) in R [37]. All transcripts in Homo Sapiens NCBI build 37.2 were used as background and Benjamini-Hochberg based FDR threshold of 0.05 was used to select significant GO biological process terms and KEGG pathways for each DE gene list [38]. We visualized the top 20 enriched KEGG pathways and GO terms in a bar chart using the lattice library (version 0.20–34) in R. We computed the union of top 20 enriched KEGG pathways/GO terms and displayed the significant FDR values (i.e., ≤ 0.05) of these pathways/terms in the three conditions.

### Network enrichment analysis of DE genes

Reactome Functional Interaction (FI) plugin in Cytoscape was used to study the known functional interactions among the DE genes [39]. First, we imported the DE genes to build a network of known functional interactions among the DE genes using the most recent Reactome FI Network annotation (version 2015). In this network, each node is a DE gene and edges represent the known functional interactions. We filtered the network by removing computationally predicted interactions and genes with zero degree (i.e., did not have any functional interaction to any other gene in the network). We clustered nodes into modules using “Cluster FI Network” function in Reactome FI plugin. We performed GO biological process enrichment on modules of size ≥ 10 and determined a representative GO term for each module by examining the GO enrichment results manually. Finally, we edited the network by setting the layout to “grouping by module ID”, labeling each module by its GO term, coloring nodes based on their upregulation/downregulation status, and adjusting the size of nodes and the font size of node labels proportional to their degree (i.e., number of interactions that they have).

### Availability of RNAseq data

RNAseq data are available for download from the National Center for Biotechnology Information Sequence Read Archive database (Accession Number: SRP102205).

## Results

### Establishment of lipid raft and nuclear localized STK4 prostate cancer cell models

To better understand the effects of STK4 enriched in the cytoplasm, lipid raft and nucleus on cell growth and gene expression in PC, first we established the Tet-inducible lipid raft (LR)- and nuclear (NL)-localized STK4 expressing C4-2 cell models in addition to the cytoplasm (CL)-localized STK4 expressing C4-2 cell model. Previously, we described the establishment of CL-localized STK4 C4-2 cell, which was included as a control in this study [27]. The Tet-inducible system allowed us to control STK4 expression in the cell. We designated these cell models as C4-2/CL-STK4, C4-2/LR-STK4, and C4-2/NL-STK4 cells (Fig 1A–1C). In this study, we utilized the C4-2 cell line because it is the castration-resistant subline of LNCaP cells and expresses significantly lower levels of STK4 transcript and protein than parental LNCaP [24]. Ectopic expression of STK4 protein in the engineered C4-2 cells was analyzed by Western blotting (Fig 1A–1D). First, we showed that doxycycline (Dox, a tetracycline analog) exposure increased the expression of ectopic CL-STK4, LR-STK4, and NL-STK4 in a time-dependent manner relative to the no Dox treatment (Fig 1A). Second, we demonstrated that ectopic STK4 protein was primarily enriched in the intended subcellular locations: cytoplasm in C4-2/CL-STK4 cell (Fig 1B), lipid raft in C4-2/LR-STK4 (Fig 1C), and nucleus in C4-2/NL-STK4 (Fig 1D) cells. Here, it is worth mentioning that the cleavage of ectopic STK4 protein (i.e. STK4-N) due to the overexpression was not detectible by Western blotting under these experimental conditions (not shown). In addition, we performed immunofluorescence imaging to verify the subcellular localization of ectopic CL-STK4, LR-STK4, and NL-STK4 protein (Fig 1E, 1F and 1G, respectively).

### STK4 enriched in cytoplasm, lipid raft, and nucleus differentially regulates cell growth

**Fig 2:**
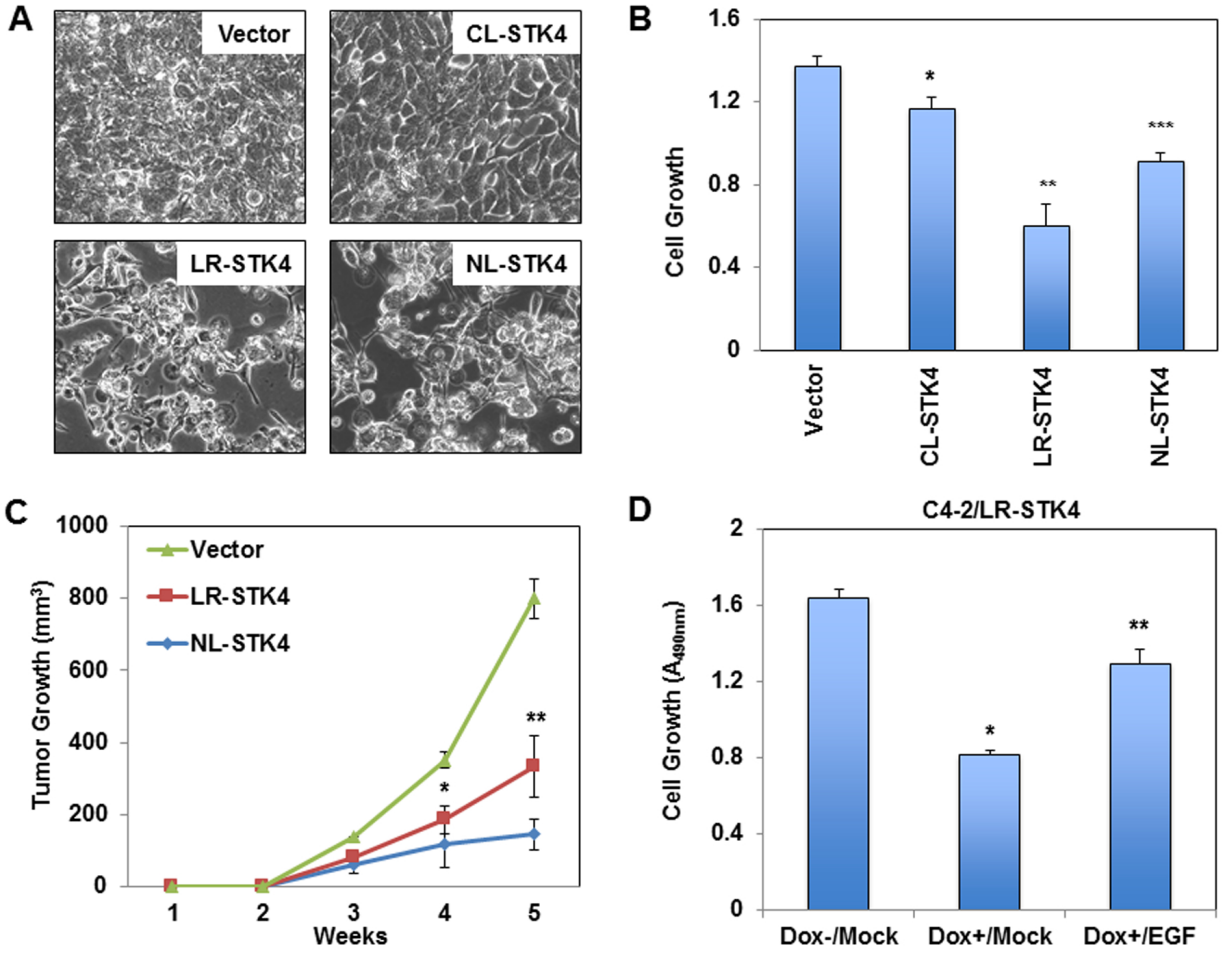
Regulation of C4-2 cell growth by STK4 signaling in all three subcellular locations. **(A)** Representative bright field images of C4-2/Vector, C4-2/CL-STK4, C4-2/LR-STK4, and C4-2/NL-STK4 cells. Cell images were captured at 72h post to Dox treatment (4 μg/ml). (**B)** Growth of C4-2/Vector, C4-2/CL-STK4, C4-2/LR-STK4, and C4-2/NL-STK4 cells *in vitro*. Cell growth was determined by MTS assay at 72h post Dox exposure. Data (±SD) are the representation of two independent experiments in triplicates, *, **, ***P < 0.007. (**C)** Prostate tumor xenografts in mice (n = 10 per conditions). C4-2/Vector, C4-2/LR-STK4, and C4-2/NL-STK4 cells were subcutaneously inoculated into the intact nude, immunocompromised male mice. Animals were treated with Dox (0.5 mg/ml) for 6 weeks in drinking water. Tumor sizes were measured weekly for 5 weeks. Tumor growth (volumes) was presented as a function of time, *, **P < 0.*01*. (**D)** Growth of C4-2/LR-STK4 cells treated with and without Dox and epidermal growth factor (EGF). Cell growth was determined by MTS assay at 72h post Dox and/or EGF treatment, *, **P < 0.*001*. Data (±SD) are the representation of two independent experiments in triplicates.

To determine whether cytoplasmic-, lipid raft-, and nuclear STK4 distinctly regulate cell growth *in vitro*, C4-2/CL-STK4, C4-2/LR-STK4, C4-2/NL-STK4 and C4-2/Vector (mock) cells were exposed to Dox to induce ectopic STK4 expression in the cell. C4-2/CL-STK4 and C4-2/Vector were included as a positive and negative control, respectively, to accurately evaluate the effects of LR-STK4 and NL-STK4 on C4-2 cell growth (Fig 2A and 2B). The results showed that the growth suppressive effects of LR-STK4 were significantly greater than NL-STK4 and CL-STK4 (*P < 0*.*01*). CL-STK4 showed the least inhibitory effects on cell growth, which is consistent with our earlier observation [27]. Therefore, the degree of growth suppression by STK4 is LR-STK4 > NL-STK4 > CL-STK4.

To determine the biological significance of the above findings, we conducted xenograft experiments in mice, according to a protocol approved by the IACUC. C4-2/LR-STK4, C4-2/NL-STK4, and C4-2/Vector cells were implanted under the skin of the hormonally intact and immunocompromised male mice (n = 10 per condition). Mice were fed with Dox in drinking water to induce STK4 expression in tumor cells. The results demonstrated that C4-2/LR-STK4 and C4-2/NL-STK4 cells formed much smaller tumors in number and size than C4-2/Vector (Fig 2C).

We noted that *in vitro* and *in vivo* growth suppression caused by the induction of LR-STK4 did not correlate. One possible explanation for it was that the growth suppressive effects of LR-STK4 might be attenuated. Evidence suggested that growth factors or cytokines could negatively regulate STK4 signaling [17, 40–42]. Indeed, treatment of LR-STK4 cells with epidermal growth factor (EGF) significantly reversed the growth inhibitory effects of LR-STK4 relative to the mock control (Fig 2D), indicating that our observations were internally consistent.

### STK4 enriched in cytoplasm, lipid raft, and nucleus differentially regulates gene expression

**Fig 3:**
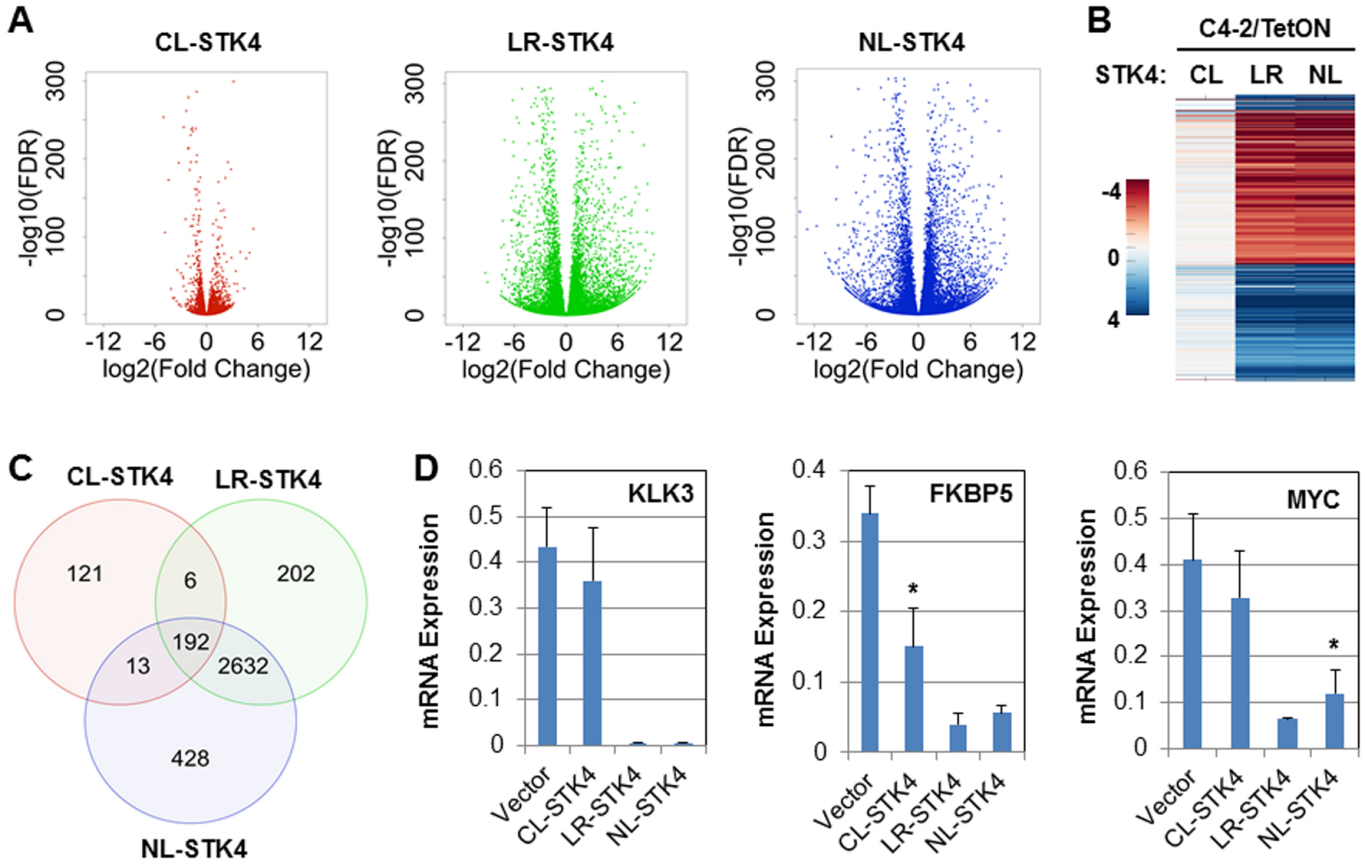
Overview of the RNAseq data and the validation. **(A)** Volcano plots of DE genes from the DESeq2 analysis. **(B)** Heatmap of DE genes using fold-change values in all three STK4 conditions (**C)** Venn diagram of DE genes in C4-2/CL-STK4, C4-2/LR-STK4, and C4-2/NL-STK4 cells. **(D)** Quantitative RT-PCR analysis of select DE genes, *P < 0.*01*. Data are representation of the two independent experiments in duplicates.

To determine whether the enrichment of STK4 signaling in the cytoplasm, lipid raft, and nucleus changes the gene expression patterns of C4-2 cells, we performed mRNA expression profiling of mock, CL-STK4, LR-STK4, and NL-STK4 cell using RNAseq (see Materials & methods). First, our statistical and bioinformatics analysis of the RNAseq data showed that the normalized gene counts between replicates were highly correlated (S1 Fig), indicating that technical variability of the sequencing between replicates were minimal. Second, we identified a list of DE genes with respect to the vector control using DESeq2. The FDR and fold change values computed by DESeq2 for all genes in all three STK4 conditions are shown in the S1 Table. To determine DE genes, we used absolute log2 fold change values ≥ 2, False Discovery Rate (FDR) ≤ 0.01 cutoffs for LR-STK4, and NL-STK4 cells. To increase the number of DE genes for CL-STK4 cells, however, we used a slightly less stringent fold change cutoff (absolute log2 fold change ≥ 1.5; FDR ≤ 0.01). The results, as illustrated in volcano plots (Fig 3A), heatmap (Fig 3B) and Venn diagram (Fig 3C) demonstrated that the number of DE genes in NL-STK4 and LR-STK4 cells were much greater than CL-STK4, and about 90% DE genes in NL-STK4 and LR-STK4 cells overlap. As detailed in the Venn diagram (Fig 3C), CL-STK4 cells resulted in 332 DE genes (226 upregulated and 106 downregulated), LR-STK4 cells resulted in 3032 DE genes (1780 upregulated and 1252 downregulated), and NL-STK4 cells resulted in 3265 DE genes (1938 upregulated and 1327 downregulated). The complete list of DE genes identified from CL-STK4, LR-STK4, and NL-STK4 cells are shown in S2–S4 Tables, respectively. The top 100 DE genes in each condition are shown in Table 1. There were 192 DE genes intersected with STK4 expression in all three subcellular compartments (Table 2). Among the intersected DE genes, seven of them were downregulated in CL-STK4, but upregulated in NL-STK4 and LR-STK4 cells, and 28 of them were upregulated in CL-STK4, but downregulated in NL-STK4 and LR-STK4 cells (Table 2). There were 2830 DE genes intersected in NL-STK4 and LR-STK4 cells (Fig 3C).

**Table 1:** Top 100 DE genes regulated by the enrichment of STK4 in three subcellular locations (*FDR ≤ 0*.*01*). Numbers are in log2 fold change. Minus signs indicate downregulation with respect to the vector control.

Entrez ID	Gene Symbol	CL-STK4	Entrez ID	Gene Symbol	LR-STK4	Entrez ID	Gene Symbol	NL-STK4
2042	EPHA3	-4.00	10417	SPON2	-8.14	158471	PRUNE2	-8.36
54898	ELOVL2	-3.41	3817	KLK2	-7.88	4311	MME	-7.19
6860	SYT4	-2.92	354	KLK3	-7.28	367	AR	-7.05
1644	DDC	2.84	26298	EHF	-6.42	8611	PPAP2A	-6.78
1836	SLC26A2	-2.78	7345	UCHL1	6.41	7345	UCHL1	6.74
55504	TNFRSF19	-2.70	158471	PRUNE2	-6.22	4128	MAOA	-6.71
7982	ST7	2.66	85414	SLC45A3	-6.16	4824	NKX3-1	-6.52
2181	ACSL3	-2.39	9415	FADS2	-5.83	999	CDH1	-6.39
2887	GRB10	2.39	2042	EPHA3	-5.75	85414	SLC45A3	-6.00
2289	FKBP5	-2.12	4824	NKX3-1	-5.72	2042	EPHA3	-5.94
64853	AIDA	1.99	999	CDH1	-5.37	9415	FADS2	-5.91
444	ASPH	1.65	8611	PPAP2A	-5.29	56937	PMEPA1	-5.57
8287	USP9Y	3.16	2346	FOLH1	-5.27	200916	RPL22L1	-5.47
58480	RHOU	-2.10	4128	MAOA	-5.17	6720	SREBF1	-5.41
79884	MAP9	-2.36	4311	MME	-5.14	2182	ACSL4	5.41
23671	TMEFF2	-4.99	154796	AMOT	4.97	261729	STEAP2	-5.31
1846	DUSP4	-2.64	2182	ACSL4	4.95	4784	NFIX	-5.14
60481	ELOVL5	-1.64	1021	CDK6	4.76	3169	FOXA1	-5.13
2982	GUCY1A3	-1.75	3169	FOXA1	-4.71	4685	NCAM2	-5.09
79993	ELOVL7	-1.58	367	AR	-4.67	154796	AMOT	5.06
22936	ELL2	-1.84	200916	RPL22L1	-4.66	6482	ST3GAL1	-4.97
1948	EFNB2	-2.07	1803	DPP4	-4.66	5567	PRKACB	-4.96
54491	FAM105A	-2.15	55504	TNFRSF19	-4.63	1803	DPP4	-4.95
563	AZGP1	-1.83	283349	RASSF3	-4.60	1021	CDK6	4.92
3158	HMGCS2	2.49	5567	PRKACB	-4.56	283349	RASSF3	-4.74
3479	IGF1	-3.24	9590	AKAP12	4.55	94241	TP53INP1	-4.74
57007	ACKR3	2.87	23052	ENDOD1	-4.38	10982	MAPRE2	4.71
4430	MYO1B	-1.96	6652	SORD	-4.37	26872	STEAP1	-4.65
6192	RPS4Y1	2.20	3315	HSPB1	-4.34	9590	AKAP12	4.62
4094	MAF	-4.42	94241	TP53INP1	-4.27	55504	TNFRSF19	-4.61
9518	GDF15	2.03	4784	NFIX	-4.27	4324	MMP15	-4.61
1026	CDKN1A	1.70	80031	SEMA6D	4.25	26084	ARHGEF26	-4.55
79822	ARHGAP28	-1.51	7782	SLC30A4	-4.23	7782	SLC30A4	-4.49
2550	GABBR1	2.15	261729	STEAP2	-4.20	3315	HSPB1	-4.48
10602	CDC42EP3	-2.44	79993	ELOVL7	-4.17	7431	VIM	4.43
4285	MIPEP	-1.52	4685	NCAM2	-4.15	439921	MXRA7	4.39
85414	SLC45A3	-1.65	26084	ARHGEF26	-4.15	6652	SORD	-4.37
56937	PMEPA1	-1.60	114569	MAL2	-4.08	10129	FRY	-4.35
9687	GREB1	-1.86	6482	ST3GAL1	-4.03	79993	ELOVL7	-4.35
120	ADD3	1.71	4436	MSH2	3.87	80031	SEMA6D	4.29
4316	MMP7	5.48	3909	LAMA3	-3.82	23052	ENDOD1	-4.29
8756	ADAM7	-4.84	5358	PLS3	3.82	3909	LAMA3	-4.27
445	ASS1	1.58	27347	STK39	-3.76	6678	SPARC	4.26
4100	MAGEA1	1.84	23705	CADM1	3.65	286077	FAM83H	-4.22
84898	PLXDC2	3.94	1534	CYB561	-3.65	9515	STXBP5L	-4.18
147381	CBLN2	5.02	25874	MPC2	-3.63	114569	MAL2	-4.11
8653	DDX3Y	2.06	10481	HOXB13	-3.62	3716	JAK1	4.06
3899	AFF3	-1.56	22998	LIMCH1	-3.59	4436	MSH2	4.00
23266	LPHN2	-2.08	11057	ABHD2	-3.58	22998	LIMCH1	-3.95
1292	COL6A2	1.53	667	DST	3.56	25874	MPC2	-3.91
1001	CDH3	2.92	22894	DIS3	3.53	11057	ABHD2	-3.82
4109	MAGEA10	4.63	2982	GUCY1A3	-3.47	22894	DIS3	3.79
4857	NOVA1	2.36	5580	PRKCD	-3.41	27347	STK39	-3.75
1135	CHRNA2	-2.40	55748	CNDP2	-3.37	25923	ATL3	-3.74
57633	LRRN1	1.73	25923	ATL3	-3.37	5358	PLS3	3.72
3397	ID1	-1.86	10257	ABCC4	-3.32	10481	HOXB13	-3.67
10551	AGR2	-3.40	57221	KIAA1244	-3.25	57683	ZDBF2	3.65
8612	PPAP2C	1.89	60481	ELOVL5	-3.23	1534	CYB561	-3.56
4233	MET	3.42	2181	ACSL3	-3.23	23705	CADM1	3.55
138046	RALYL	-2.96	2335	FN1	-3.05	667	DST	3.54
162394	SLFN5	1.58	23195	MDN1	3.02	2335	FN1	-3.44
285025	CCDC141	-2.76	11167	FSTL1	2.99	57221	KIAA1244	-3.41
54842	MFSD6	1.86	23327	NEDD4L	-2.93	55748	CNDP2	-3.39
116443	GRIN3A	1.87	6675	UAP1	-2.89	22873	DZIP1	3.35
1740	DLG2	-2.69	5836	PYGL	2.86	64780	MICAL1	-3.31
80150	ASRGL1	-1.83	55884	WSB2	-2.84	2982	GUCY1A3	-3.28
3934	LCN2	2.35	3417	IDH1	-2.81	5580	PRKCD	-3.28
4137	MAPT	1.72	288	ANK3	-2.75	7105	TSPAN6	3.25
23467	NPTXR	1.79	56994	CHPT1	-2.74	9882	TBC1D4	3.22
6091	ROBO1	-1.63	7764	ZNF217	-2.71	56204	FAM214A	-3.17
90362	FAM110B	-1.55	5530	PPP3CA	-2.70	64839	FBXL17	-3.14
4664	NAB1	2.07	55704	CCDC88A	2.69	60481	ELOVL5	-3.13
9783	RIMS3	2.37	2289	FKBP5	-2.50	5836	PYGL	3.11
389206	BEND4	-1.71	3992	FADS1	-2.43	2181	ACSL3	-3.11
6286	S100P	-2.03	79718	TBL1XR1	-2.42	5530	PPP3CA	-3.04
330	BIRC3	1.91	57222	ERGIC1	-2.39	1528	CYB5A	-3.02
5796	PTPRK	1.97	55827	DCAF6	-2.38	11167	FSTL1	3.01
5801	PTPRR	3.23	2195	FAT1	2.30	57211	GPR126	-2.96
57628	DPP10	3.56	7163	TPD52	-2.28	51347	TAOK3	-2.95
51351	ZNF117	2.05	8826	IQGAP1	2.25	928	CD9	-2.88
79674	VEPH1	-2.65	64062	RBM26	2.24	288	ANK3	-2.85
64881	PCDH20	2.63	2194	FASN	-2.22	22936	ELL2	-2.85
2026	ENO2	1.95	23013	SPEN	2.19	6091	ROBO1	2.84
10133	OPTN	2.20	29968	PSAT1	-2.10	23327	NEDD4L	-2.83
5066	PAM	1.87	6747	SSR3	-2.03	4942	OAT	2.82
7102	TSPAN7	4.40	6678	SPARC	4.19	7764	ZNF217	-2.81
80031	SEMA6D	-4.13	51347	TAOK3	-3.23	56994	CHPT1	-2.80
26053	AUTS2	1.78	9882	TBC1D4	3.10	55884	WSB2	-2.79
284119	PTRF	2.13	56937	PMEPA1	-5.13	57619	SHROOM3	-2.74
162514	TRPV3	-2.41	6091	ROBO1	2.79	6675	UAP1	-2.70
5268	SERPINB5	1.65	159195	USP54	-2.51	55704	CCDC88A	2.70
9644	SH3PXD2A	1.59	2235	FECH	-2.55	3417	IDH1	-2.69
55243	KIRREL	3.62	57211	GPR126	-2.86	1174	AP1S1	-2.69
4117	MAK	-1.67	57683	ZDBF2	3.45	79718	TBL1XR1	-2.63
3371	TNC	1.71	2131	EXT1	3.50	1946	EFNA5	-2.63
57562	KIAA1377	2.32	7431	VIM	4.54	5768	QSOX1	-2.62
10158	PDZK1IP1	1.51	440	ASNS	-2.34	159195	USP54	-2.61
9086	EIF1AY	1.85	23657	SLC7A11	-3.54	51166	AADAT	-2.60
8284	KDM5D	1.64	51635	DHRS7	-2.74	57222	ERGIC1	-2.54
5352	PLOD2	1.57	595	CCND1	-2.25	3304	HSPA1B	2.52

**Table 2:** Common DE genes regulated by the enrichment of STK4 in three subcellular locations *(FDR ≤ 0*.*01)*. Numbers are in log2 fold change. Minus signs indicate downregulation with respect to the vector control.

Entrez ID	Gene Symbol	CL-STK4	LR-STK4	NL-STK4	Entrez ID	Gene Symbol	CL-STK4	LR-STK4	NL-STK4
23316	CUX2	-1.61	2.64	2.8	3113	HLA-DPA1	2.03	4.98	4.8
3087	HHEX	-2.09	2.33	2.4	3239	HOXD13	1.70	7.53	7.6
3400	ID4	-2.00	3.57	3.5	11255	HRH3	-1.53	-3.61	-3.8
5064	PALM	-2.19	2.76	2.8	9951	HS3ST4	-3.12	-4.71	-5.3
6091	ROBO1	-1.63	2.79	2.8	3434	IFIT1	1.86	2.86	2.7
80031	SEMA6D	-4.13	4.25	4.3	3479	IGF1	-3.24	-6.94	-8.7
57713	SFMBT2	-1.60	4.41	4.4	3751	KCND2	-1.58	-3.06	-3.7
57007	ACKR3	2.87	-2.45	-5.7	57562	KIAA1377	2.32	3.17	2.9
6364	CCL20	1.74	-3.12	-4.5	3798	KIF5A	2.68	6.71	6.6
51816	CECR1	2.08	-2.60	-2.6	55243	KIRREL	3.62	6.78	6.8
170712	COX7B2	2.12	-2.71	-3.9	84894	LINGO1	1.84	3.70	3.9
1644	DDC	2.84	-3.41	-7.1	54947	LPCAT2	1.92	4.68	5.0
8653	DDX3Y	2.06	-4.39	-7.1	84230	LRRC8C	1.99	5.70	5.6
9086	EIF1AY	1.85	-3.23	-5.2	130576	LYPD6B	2.36	2.40	3.1
9518	GDF15	2.03	-6.69	-7.5	4117	MAK	-1.67	-2.40	-2.3
219970	GLYATL2	1.55	-3.86	-6.0	22983	MAST1	1.50	2.29	2.3
3158	HMGCS2	2.49	-3.53	-7.1	154141	MBOAT1	1.71	4.01	4.1
153572	IRX2	1.76	-3.08	-3.6	2122	MECOM	2.76	5.13	5.0
8284	KDM5D	1.64	-3.18	-5.7	4233	MET	3.42	6.10	6.2
3934	LCN2	2.35	-3.90	-4.9	11320	MGAT4A	2.14	3.91	3.9
57633	LRRN1	1.73	-4.01	-6.5	4281	MID1	2.09	7.94	7.8
4100	MAGEA1	1.84	-3.60	-7.4	4285	MIPEP	-1.52	-2.76	-2.4
4316	MMP7	5.48	-3.03	-3.6	26002	MOXD1	2.85	7.08	7.6
22829	NLGN4Y	1.53	-3.68	-4.3	4664	NAB1	2.07	3.12	3.3
10158	PDZK1IP1	1.51	-4.07	-6.6	57701	NCKAP5L	1.62	2.41	2.6
5284	PIGR	1.82	-2.72	-2.8	23114	NFASC	1.57	2.58	2.3
8612	PPAP2C	1.89	-3.94	-5.9	7025	NR2F1	1.61	3.62	3.3
64063	PRSS22	1.73	-3.55	-4.1	2908	NR3C1	2.58	8.50	8.3
5696	PSMB8	2.53	-2.10	-2.6	4922	NTS	2.08	4.71	4.7
6192	RPS4Y1	2.20	-3.63	-7.8	93145	OLFM2	2.42	2.62	2.5
5268	SERPINB5	1.65	-4.31	-5.5	10133	OPTN	2.20	2.02	2.1
8764	TNFRSF14	1.62	-3.95	-4.2	390190	OR5B2	-2.17	-3.93	-4.5
7367	UGT2B17	1.63	-2.16	-4.7	5066	PAM	1.87	3.88	4.0
8287	USP9Y	3.16	-2.47	-6.6	64881	PCDH20	2.63	2.51	2.9
7404	UTY	2.08	-2.79	-4.9	56034	PDGFC	2.02	6.40	6.4
728763	AC104809.3	-1.89	-4.27	-4.9	114770	PGLYRP2	-2.23	-5.75	-6.8
2181	ACSL3	-2.39	-3.23	-3.1	5241	PGR	2.90	6.04	6.1
23305	ACSL6	1.75	3.86	3.6	5569	PKIA	2.53	5.84	5.9
10863	ADAM28	-2.48	-4.17	-3.6	51365	PLA1A	-2.18	-5.94	-6.6
8756	ADAM7	-4.84	-7.51	-8.2	440107	PLEKHG7	-1.51	-3.55	-4.1
150	ADRA2A	-1.72	-4.08	-5.9	5352	PLOD2	1.57	3.94	4.3
100130776	AGAP2-AS1	1.58	3.05	3.4	84898	PLXDC2	3.94	3.66	3.4
10551	AGR2	-3.40	-7.05	-7.1	56937	PMEPA1	-1.60	-5.13	-5.6
183	AGT	-1.96	-4.95	-5.1	57718	PPP4R4	1.93	2.56	2.8
57491	AHRR	1.52	5.95	6.2	5743	PTGS2	1.53	2.96	3.8
115701	ALPK2	-2.23	-3.52	-3.7	5796	PTPRK	1.97	3.36	3.3
347902	AMIGO2	1.83	3.16	3.3	284119	PTRF	2.13	2.65	2.9
319	APOF	-1.82	-4.53	-4.7	138046	RALYL	-2.96	-6.93	-7.6
79822	ARHGAP28	-1.51	-2.60	-2.6	11069	RAPGEF4	1.51	4.05	4.2
115557	ARHGEF25	1.81	2.30	2.4	85004	RERG	2.02	3.99	4.3
26053	AUTS2	1.78	3.23	3.1	10287	RGS19	1.70	3.28	3.3
563	AZGP1	-1.83	-7.37	-10.2	58480	RHOU	-2.10	-2.27	-2.4
25825	BACE2	1.98	3.56	3.5	9783	RIMS3	2.37	2.45	2.4
54796	BNC2	2.07	4.92	4.9	140730	RIMS4	1.51	6.12	6.2
89927	C16orf45	1.92	2.32	2.2	57484	RNF150	1.54	4.52	4.6
767	CA8	3.00	6.92	7.3	221687	RNF182	2.10	3.82	3.8
57118	CAMK1D	1.91	3.68	3.9	6286	S100P	-2.03	-6.00	-5.5
858	CAV2	3.15	6.26	6.5	55511	SAGE1	-2.23	-4.94	-5.6
285025	CCDC141	-2.76	-4.30	-4.8	389432	SAMD5	2.55	3.04	3.0
159989	CCDC67	-2.41	-4.89	-5.5	54809	SAMD9	2.68	3.25	3.8
151887	CCDC80	2.66	5.68	5.3	6326	SCN2A	2.46	4.71	4.6
9308	CD83	1.68	2.84	2.7	56256	SERTAD4	2.46	6.28	6.1
64781	CERK	2.54	5.64	5.7	9644	SH3PXD2A	1.59	3.65	3.8
1135	CHRNA2	-2.40	-7.59	-8.3	6565	SLC15A2	-1.65	-2.46	-2.1
9435	CHST2	2.14	4.65	4.9	7781	SLC30A3	2.20	5.05	5.0
7123	CLEC3B	-1.51	-3.95	-4.1	55089	SLC38A4	-3.73	-2.73	-2.1
80034	CSRNP3	2.12	3.16	3.2	85414	SLC45A3	-1.65	-6.16	-6.0
1519	CTSO	-1.57	-2.58	-2.2	146857	SLFN13	2.50	3.17	3.0
10563	CXCL13	-2.96	-4.32	-4.4	84189	SLITRK6	-1.68	-3.89	-3.5
80319	CXXC4	2.35	5.90	5.8	8406	SRPX	2.25	2.73	2.8
260293	CYP4X1	2.04	2.66	3.1	7903	ST8SIA4	2.61	2.73	3.4
1740	DLG2	-2.69	-3.44	-3.8	6769	STAC	2.32	4.58	4.6
93099	DMKN	1.60	5.54	5.8	112755	STX1B	1.83	4.45	4.9
10655	DMRT2	-1.69	-3.91	-4.0	55061	SUSD4	1.54	2.47	3.0
1846	DUSP4	-2.64	-3.71	-3.5	221711	SYCP2L	1.75	4.82	4.8
80303	EFHD1	1.50	4.55	4.8	23345	SYNE1	1.90	4.46	4.4
22936	ELL2	-1.84	-3.10	-2.8	11346	SYNPO	1.52	2.86	2.7
54898	ELOVL2	-3.41	-2.33	-2.1	6857	SYT1	1.72	4.63	5.0
60481	ELOVL5	-1.64	-3.23	-3.1	6860	SYT4	-2.92	-6.38	-8.5
79993	ELOVL7	-1.58	-4.17	-4.3	80731	THSD7B	-1.91	-3.73	-4.0
2026	ENO2	1.95	3.18	3.4	23671	TMEFF2	-4.99	-6.34	-6.3
2042	EPHA3	-4.00	-5.75	-5.9	55321	TMEM74B	2.55	3.90	3.7
2119	ETV5	1.88	3.40	4.3	3371	TNC	1.71	4.63	3.9
90362	FAM110B	-1.55	-2.47	-2.4	55504	TNFRSF19	-2.70	-4.63	-4.6
9715	FAM131B	1.85	3.45	4.1	23043	TNIK	1.64	3.97	3.8
23359	FAM189A1	2.02	2.02	2.0	84951	TNS4	-1.94	-5.00	-5.3
2217	FCGRT	2.29	4.06	4.2	10345	TRDN	-1.76	-5.87	-6.3
9638	FEZ1	1.90	2.14	2.8	117854	TRIM6	1.74	4.92	5.0
2289	FKBP5	-2.12	-2.50	-2.3	7220	TRPC1	1.89	6.36	6.6
121643	FOXN4	2.14	2.19	2.2	162514	TRPV3	-2.41	-3.49	-3.7
53827	FXYD5	2.27	6.68	7.0	10100	TSPAN2	1.78	5.64	6.1
2550	GABBR1	2.15	2.39	2.2	7102	TSPAN7	4.40	5.84	5.8
2706	GJB2	1.72	2.16	3.1	23508	TTC9	1.55	2.72	2.7
647309	GMNC	-1.95	-3.46	-5.3	10382	TUBB4A	1.52	2.80	2.8
387509	GPR153	1.50	3.25	3.1	54490	UGT2B28	-2.08	-4.54	-5.2
2982	GUCY1A3	-1.75	-3.47	-3.3	79674	VEPH1	-2.65	-5.42	-6.2

To validate our RNAseq data, we analyzed the levels of KLK3, FKBP5, and MYC mRNA expression in the engineered C4-2 cells. We selected these genes because (i) their expressions were differentially regulated by STK4 in all three conditions (S1 Table), (ii) KLK3 and FKBP5 are well-known targets of AR that is also negatively regulated by STK4 [43] and (iii) MYC is a YAP/TEAD target and it intersected with STK4 signaling in PC cells [24]. Our RNAseq data revealed that expression of KLK3 was inhibited 7-fold in LR-STK4 and 13-fold in NL-STK4 cells. Similarly, expression of FKBP5 was inhibited 2-fold in CL-STK4, 2.5-fold in LR-STK4, and 2.3-fold in NL-STK4 cells. In addition, expression of MYC was inhibited 1.6-fold in LR-STK4 and NL-STK4 cells. These fold change values are in log2. Induction of CL-STK4 inhibited the expression of KLK3 and MYC less than half fold. Our quantitative PCR analysis verified the RNAseq data that, indeed, STK4 expression in all three cell compartments differentially regulates the expression of KLK3, FKBP5, and MYC (Fig 3D). We also examined the impact of STK4 on the AR pathway genes (Table 3) obtained from Wikipathways [44]. The results showed that several AR targets were upregulated or downregulated by STK4 expression in these subcellular compartments, further validating our RNAseq data.

**Table 3:** Fold change values of AR targets in response to STK4 enrichment in all three subcellular compartments *(FDR ≤ 0*.*01)*. Fold change values are in log2. Minus signs indicate downregulation with respect to the vector control.

Entrez ID	Gene Symbol	CL-STK4	LR-STK4	NL-STK4	Entrez ID	Gene Symbol	CL-STK4	LR-STK4	NL-STK4
367	AR	0.28	-4.67	-7.05	9612	NCOR2	0.09	-0.97	-0.99
595	CCND1	-0.05	-2.25	-2.04	1499	CTNNB1	0.15	0.41	0.43
8611	PPAP2A	-0.18	-5.29	-6.78	860	RUNX2	-0.71	-1.48	-1.74
57178	ZMIZ1	0.24	-1.71	-1.81	5925	RB1	0.44	0.61	0.75
23028	KDM1A	-0.34	1.06	1.28	8648	NCOA1	-0.02	-0.55	-0.63
3725	JUN	0.25	2.52	2.54	10401	PIAS3	0.43	-0.72	-0.74
1956	EGFR	0.77	-2.41	-2.36	1387	CREBBP	-0.29	0.53	0.53
4088	SMAD3	0.93	2.94	2.95	2119	ETV5	1.88	3.40	4.32
998	CDC42	-0.14	1.64	1.75	898	CCNE1	-0.14	0.47	0.80
354	KLK3	-0.71	-7.28	-13.71	2033	EP300	-0.12	0.35	0.35
24149	ZNF318	0.34	1.44	1.48	29893	PSMC3IP	-0.31	0.61	0.85
811	CALR	-0.06	-1.49	-1.11	10499	NCOA2	0.13	-0.46	-0.47
1026	CDKN1A	1.70	-2.11	-1.95	6714	SRC	0.05	-0.66	-0.61
573	BAG1	0.22	-2.03	-1.78	5728	PTEN	0.20	0.54	0.44
6093	ROCK1	-0.01	0.98	1.12	11034	DSTN	-0.10	0.61	0.54
5901	RAN	-0.39	0.64	0.72	90427	BMF	-0.35	-0.54	-0.90
11143	KAT7	0.05	0.91	1.21	2288	FKBP4	-0.10	-0.31	-0.23
9475	ROCK2	0.20	0.91	1.05	23598	PATZ1	-0.03	0.45	0.53
207	AKT1	0.16	-1.03	-1.11	64800	EFCAB6	0.27	2.63	2.62
9611	NCOR1	0.33	0.69	0.86	7041	TGFB1I1	0.40	1.79	3.00
2308	FOXO1	-0.26	1.58	1.85	11315	PARK7	-0.22	-0.21	-0.21
2932	GSK3B	0.03	-0.79	-0.92	1385	CREB1	-0.10	-0.37	-0.43
6774	STAT3	0.24	-1.06	-0.90	25942	SIN3A	0.19	0.08	0.19
2274	FHL2	-0.34	3.70	3.66	8554	PIAS1	-0.53	-0.38	-0.26
672	BRCA1	-0.26	0.83	0.97	3065	HDAC1	-0.11	0.05	0.15
387	RHOA	0.06	0.54	0.57	7329	UBE2I	-0.04	-0.19	-0.19
7341	SUMO1	-0.13	0.80	0.87	6667	SP1	0.17	0.33	0.17
8850	KAT2B	0.21	1.20	1.28	6013	RLN1	0.36	-2.68	-2.21
10273	STUB1	0.11	-1.77	-1.77	4193	MDM2	0.42	-0.10	-0.16
5052	PRDX1	0.31	0.56	0.65	5970	RELA	0.26	0.01	0.16
5295	PIK3R1	0.12	-1.06	-1.39	5879	RAC1	-0.10	0.03	0.10
6049	RNF6	-0.15	-1.17	-0.86	388	RHOB	0.42	0.22	0.17
7337	UBE3A	-0.13	-0.82	-0.68	5296	PIK3R2	-0.22	-0.78	-1.21
5747	PTK2	-0.10	0.66	0.61	9604	RNF14	-0.17	0.00	-0.07
166	AES	0.24	-0.84	-0.77	8202	NCOA3	0.30	-0.08	0.06
23411	SIRT1	-0.33	0.97	1.00	5883	RAD9A	0.24	0.12	-0.10
10399	GNB2L1	0.17	0.37	0.36	56924	PAK6	0.36	-1.07	0.55
857	CAV1	1.09	6.33	6.42	8431	NR0B2	0.14	0.84	0.69
3985	LIMK2	-0.39	-0.73	-0.85	10524	KAT5	0.11	-0.24	-0.09
6605	SMARCE1	-0.05	0.71	0.93	6047	RNF4	-0.05	-0.19	-0.04
1616	DAXX	0.12	0.67	0.66	4089	SMAD4	0.06	0.04	-0.03
7050	TGIF1	0.24	-1.23	-1.24	7182	NR2C2	0.15	-0.01	-0.03
9063	PIAS2	0.24	-0.98	-0.84	51588	PIAS4	0.01	0.29	-0.03
10498	CARM1	0.09	0.93	0.83	2316	FLNA	-0.19	-0.03	0.01

**Fig 4:**
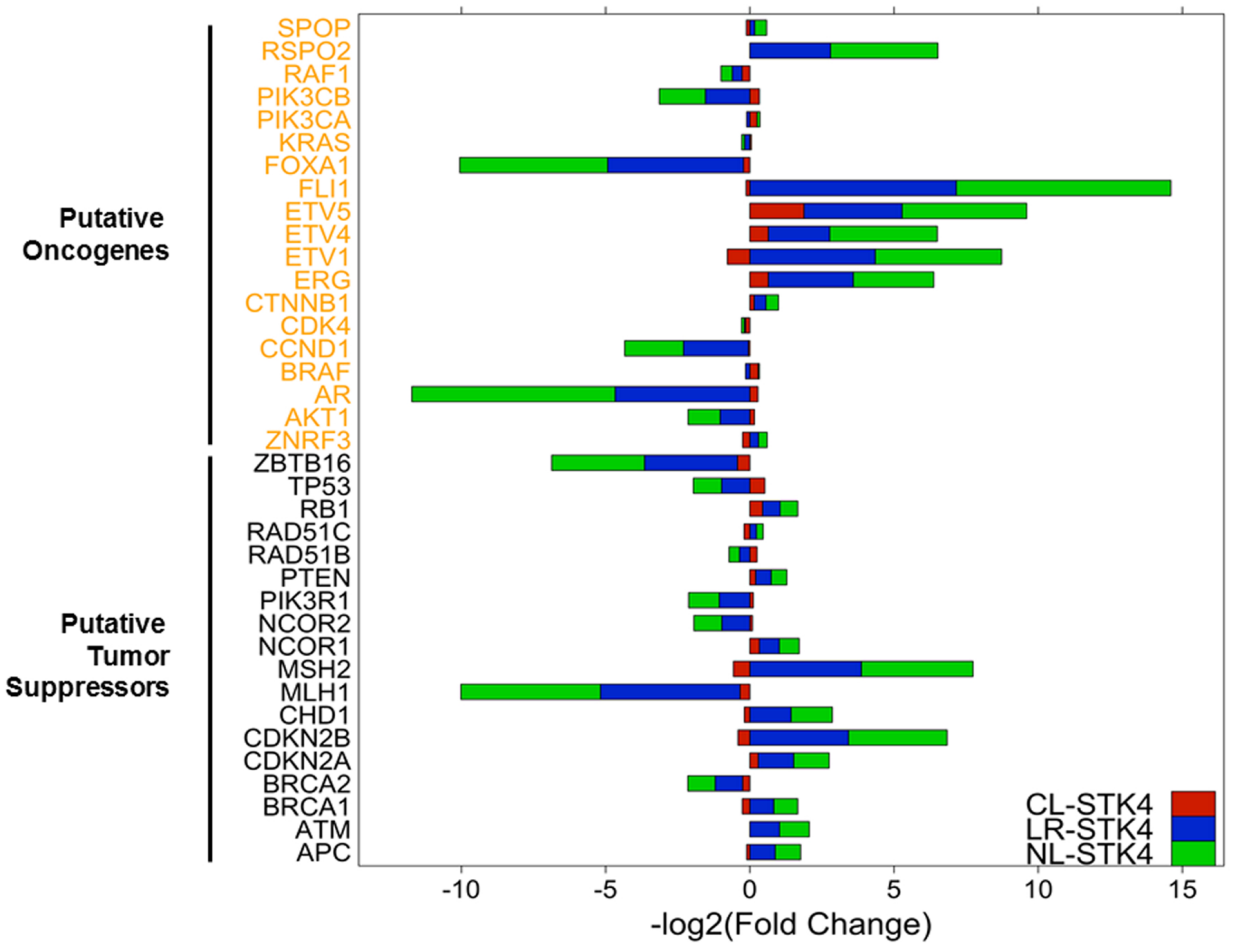
STK4 selectively regulates the expression of putative oncogenes and tumor suppressors. Log2 fold change values for a list of putative oncogenes (black) and tumor suppressors (orange) in CL-STK4, LR-STK4, and NL-STK4 cells.

In addition, we compared our DE genes to the known putative oncogenes and tumor suppressors [45, 46]. Fig 4 shows that enrichment of STK4 in all three subcellular compartments selectively regulated the expression of putative oncogenes and tumor suppressors. Many of these genes are directly linked to the AR pathway (e.g. FOXA1, SPOP, NCOR1/2, and ZBTB16), the DNA repair mechanism (e.g. MLH1 and MSH2), a member of the EST factors (e.g. ERG, ETV1/4/5, and FLH1), and cycle regulators (e.g. CDKN1A and CDKN2B). These genes and pathways are suggested to play a critical role in PC biology including metastatic CRPC [45, 46].

### DE genes regulated by STK4 signaling are associated with multiple biological pathways

**Fig 5:**
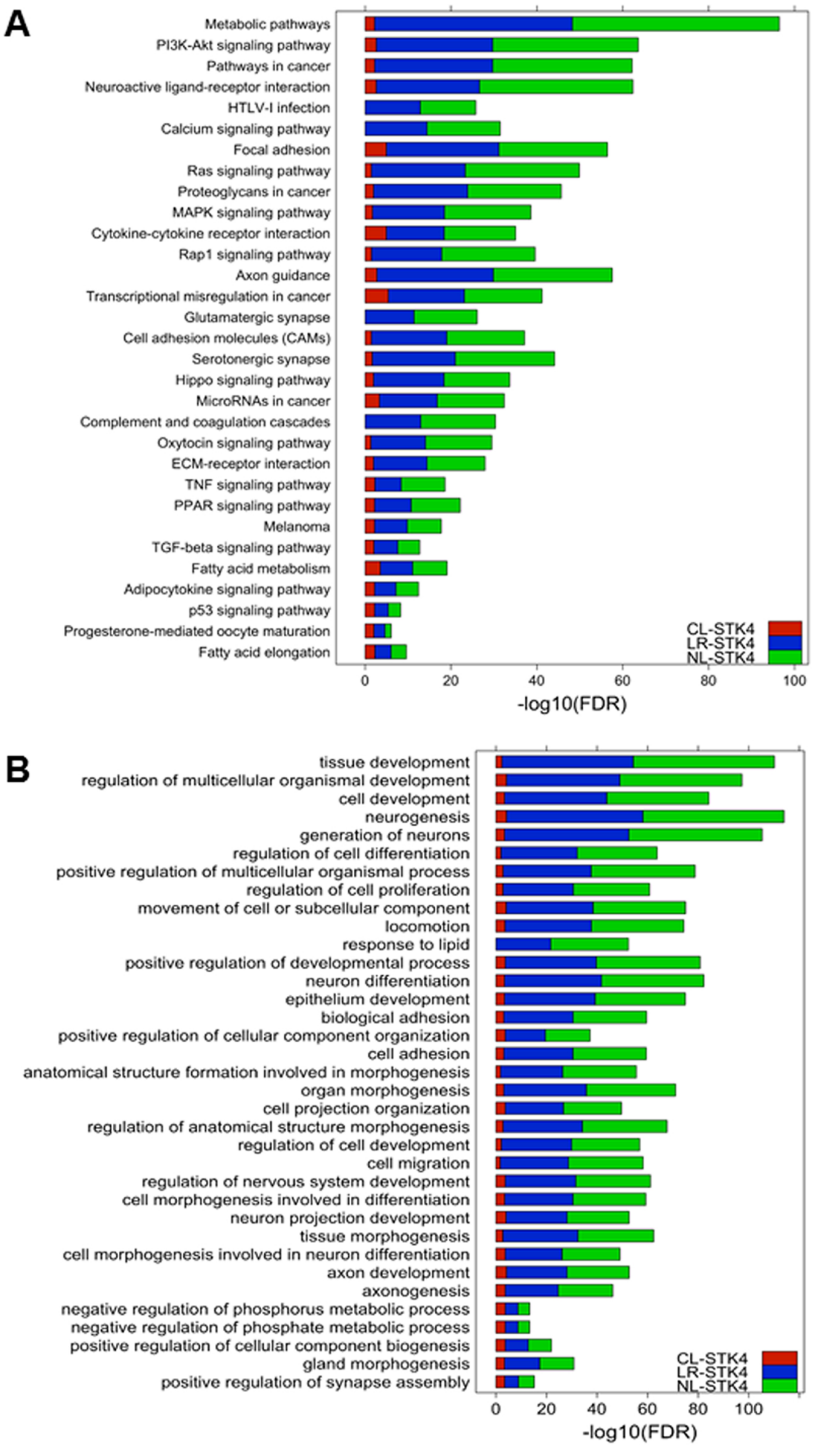
Top 20 KEGG pathways and GO biological process terms enriched by DE genes in all three STK4 cells. Each term in the bar chart appears in the top 20 **(A)** KEGG pathways **(B)** GO biological process terms enriched by DE genes in at least one condition. Red, blue, and green bars refer to the log2 enrichment FDR values in CL-STK4, LR-STK4, and NL-STK4, respectively. The terms with missing FDR values for CL-STK4 indicate that the DE genes in CL-STK4 were not significantly enriched for those terms.

To gain insights into the biological processes and pathways associated with DE genes, we performed KEGG pathway and Gene Ontology (GO) biological processes enrichment analysis using STRINGdb package in R [37]. We found 45, 214, and 216 KEGG pathways enriched by DE genes in CL-STK4, LR-STK4, and NL-STK4 cells, respectively (S5–S7 Tables). There were eight and five KEGG pathways uniquely enriched in LR-STK4 and NL-STK4 cells, respectively. On the other hand, there were no KEGG pathways uniquely enriched in CL-STK4 cells. Top 20 enriched KEGG pathways are shown in Fig 5A and the complete results are shown in S5–S7 Tables. The KEGG pathway enrichment results show that majority of the top enriched pathways (e.g., Metabolic pathways, PI3K-AKT signaling, Pathways in cancer, Focal adhesion, Axon guidance, MAPK signaling, and Fatty acid metabolism) are common in all three conditions. Due to the large number of DE genes in NL-STK4 and LR-STK4 conditions, GO enrichment analysis returned over 2,000 terms. Top 20 enriched GO biological process terms are shown in Fig 5B and complete results are shown in S8–S10 Tables. Top GO terms included terms such as development, proliferation, differentiation, axonogenesis, metabolic process, and cell adhesion.

**Fig 6:**
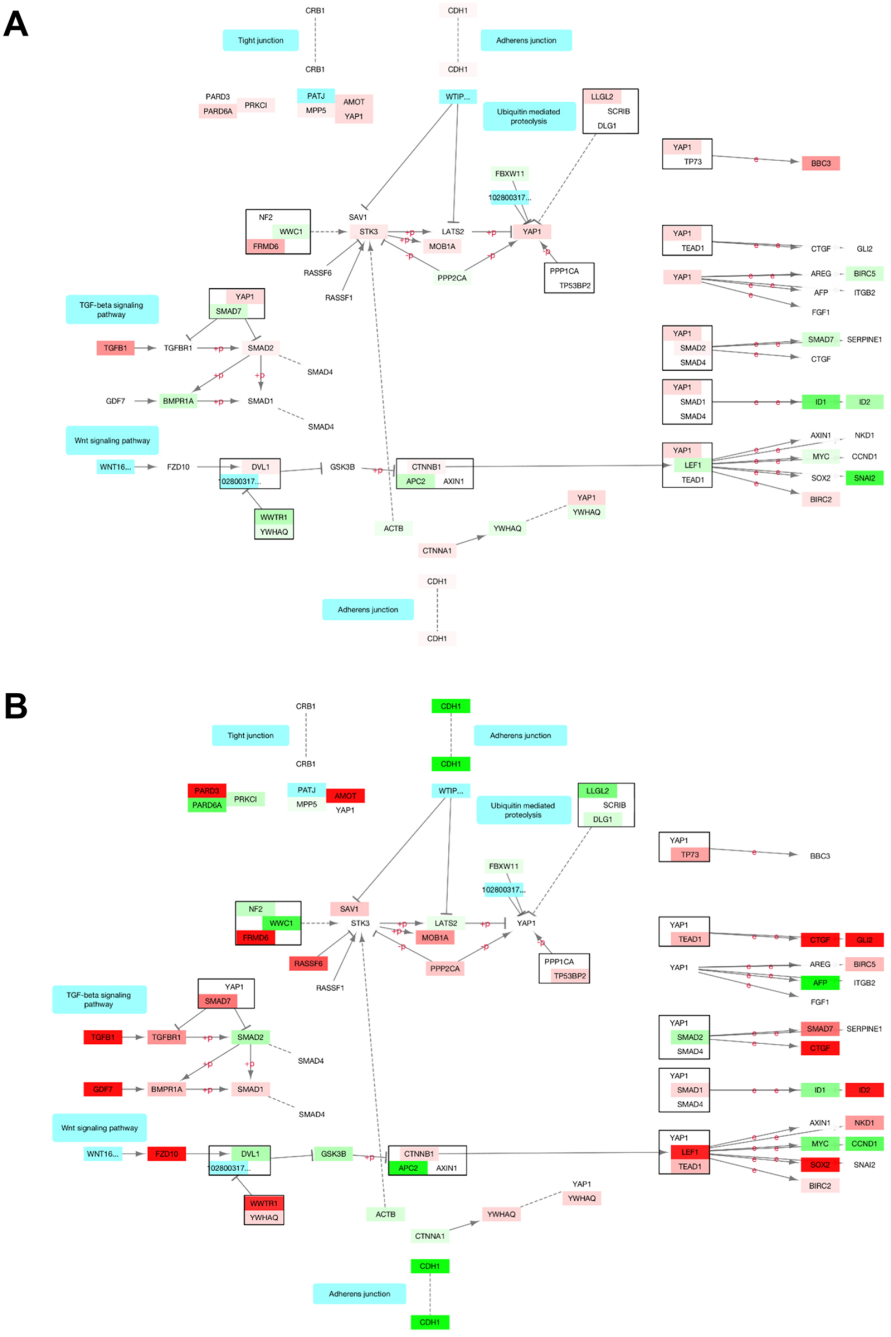
KEGG Hippo signaling pathway. Each gene is colored by gene expression fold change in (A) CL-STK4 (B) NL-STK4 conditions using Cytoscape. Red: Upregulated, green: downregulated, white: not differentially expressed. Edge labels “e”: expression interaction, “+p”: phosphorylation, “-p”: dephosphorylation.

In addition, because STK4 is a potent inhibitor of the YAP1/WWTR1-dependent transcriptions, we evaluated the status of YAP1/WWTR1 targets with respect to the subcellular localization of STK4 (S11 Table). We obtained 48 YAP1/WWTR1 targets from published studies [47–50]. We also illustrated the expression status of the Hippo pathway components in CL-STK4 (Fig 6A), NL-STK4 (Fig 6B), and LR-STK4 (S2 Fig) cells. The results of these investigations demonstrated that CL-STK4 modestly and distinctly altered the expression of YAP1/WWTR1 targets and the Hippo pathway members compared with LR-STK4 and NL-STK4. For example, CL-STK4 slightly increased STK3 and LLGL2 transcripts while LR-STK4 and NL-STK4 downregulated LLGL2 without affecting STK3 transcripts. In addition, LR-STK4 and NL-STK4 differentially regulated YAP1/WWTR1 targets (Fig 6B). For instance, LR-STK4 downregulated YAP1 target ITGB2, whereas NL-STK4 had no effect. Likewise, CL-STK4 and LR-STK4 slightly increased YAP1 expression, but NL-STK4 did not.

### DE genes in NL-STK4 and LR-STK4 cells associate with overlapping and unique modules

**Fig 7:**
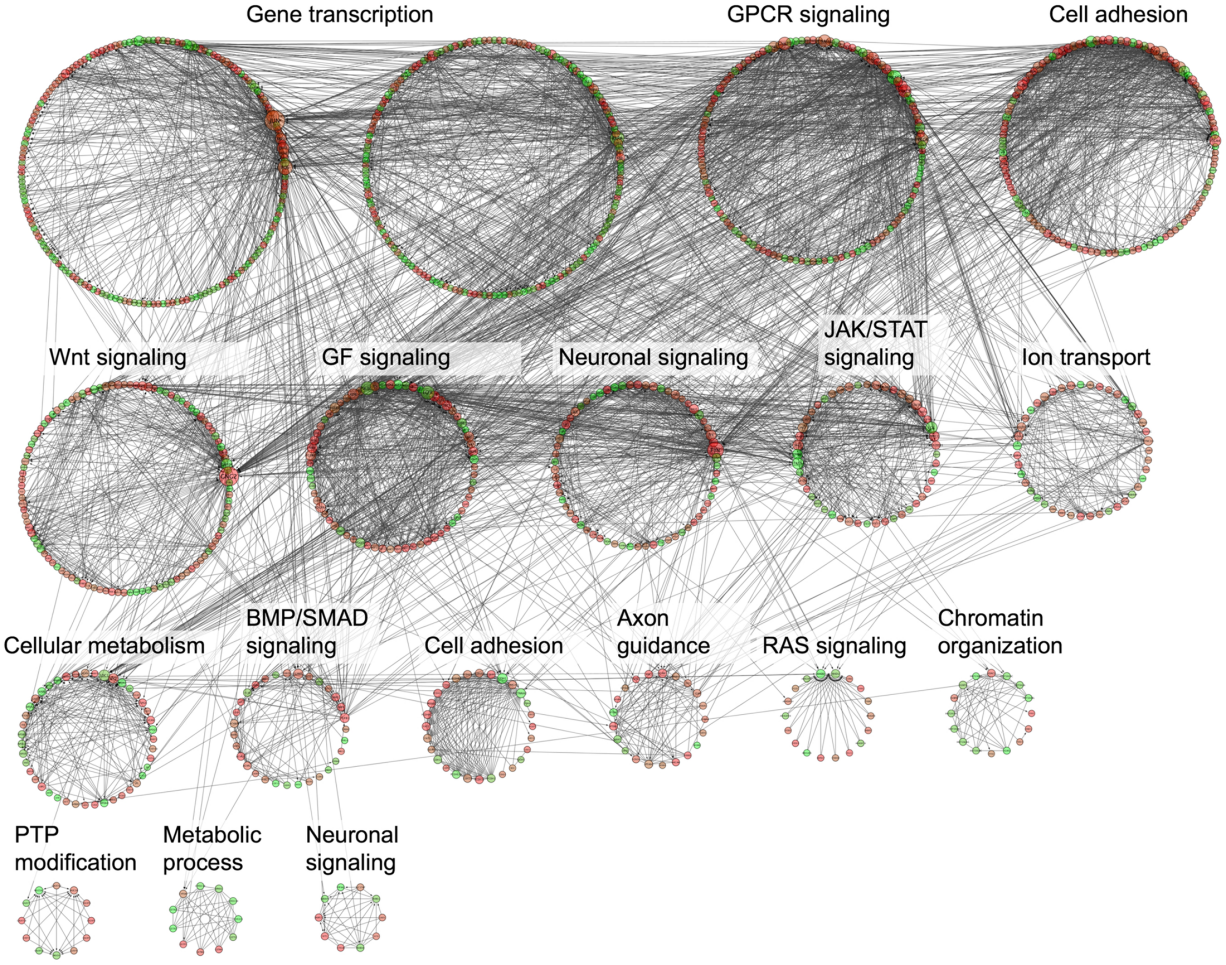
Network enrichment annotation of DE genes in NL-STK4 cells. DE genes in NL-STK4 cells that have known functional interactions were clustered based on their connectivity in the network using Reactome FI plugin in Cytoscape. Each clustered module was annotated to a representative significantly-enriched GO term. Each edge represents a known interaction between two genes (i.e., nodes) in the network.

**Fig 8:**
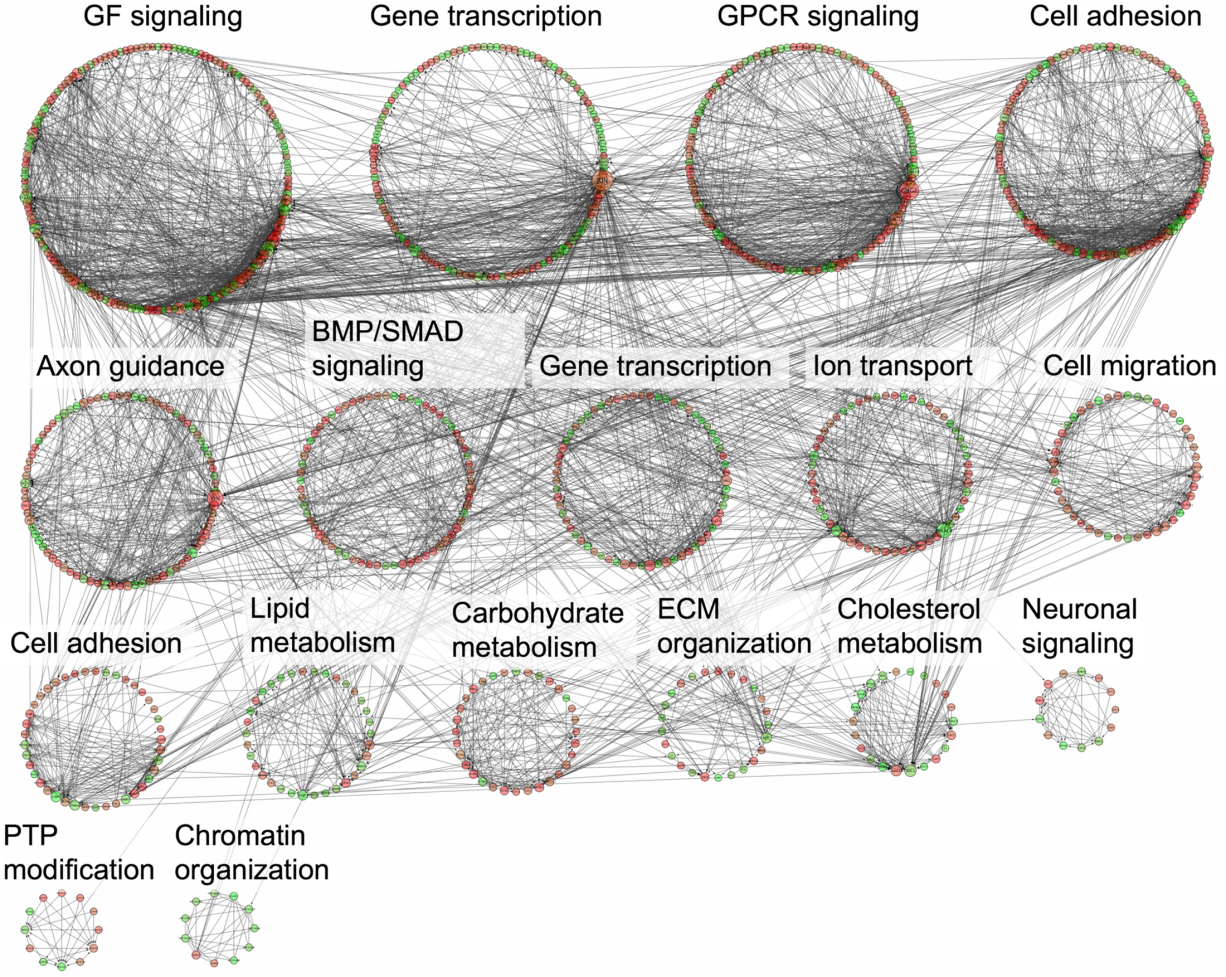
Network enrichment annotation of DE genes in LR-STK4 cells. DE genes in LR-STK4 cells that have known functional interactions were clustered based on their connectivity in the network using Reactome FI plugin in Cytoscape. Each clustered module was annotated to a representative significantly-enriched GO term. Each edge represents a known interaction between two genes (i.e., nodes) in the network.

We investigated known functional interactions between DE genes in each condition using Reactome FI plugin in Cytoscape [51]. We built an interaction network of DE genes based on known functional interactions and clustered the network into modules. We annotated each module with a significantly enriched GO term (see Materials & methods). The annotated networks of DE genes for LR-STK4 and NL-STK4 cells were shown in Figs 7 and 8, respectively. Because a few number of DE genes existed in CL-STK4 cells, the network annotation for those DE genes were not performed. The interaction network of NL-STK4 had 1086 genes grouped into 18 modules of size ≥ 10 genes. The interaction network of LR-STK4 had 974 genes grouped into 17 modules of size ≥ 10 genes. Both networks had eight common module annotations such as gene transcription, cell adhesion, and axon guidance, which also appeared in the KEGG pathway enrichment results. The NL-STK4 network had unique GO terms such as WNT signaling, RAS signaling, JAK/STAT signaling, and metabolic processes whereas the LR-STK4 network had unique GO terms such as cell migration, lipid metabolism, and ECM organization. Since clustering of the nodes were not based on GO annotation of genes, but solely based on the known functional interactions between the genes, some modules were enriched in the same GO term, namely gene transcription, cell adhesion, and axon guidance. These findings indicate that STK4/Hippo signaling initiating from or transiting through lipid raft or cell nuclei not only has overlapping functions but it also confers unique functions in regulating cell growth.

## Discussion

In this study, we have demonstrated that controlled expression of STK4, primarily enriched in the cytoplasm, lipid raft, and nucleus, differentially regulates PC cell growth and gene expression. We have identified several DE genes whose expression is downregulated or upregulated by CL-STK4, LR-STK4, and NL-STK4 signaling in all three subcellular locations. Surprisingly, about 90% of DE genes that were regulated by LR-STK4 and NL-STK4 overlapped and the number of DE genes identified from LR-STK4 and NL-STK4 cells were much greater than CL-STK4 cells. Our functional annotation clustering showed that these DE genes were associated with a broad cellular biology including cell proliferation, differentiation, motility, adhesion, survival, apoptosis, axon guidance, and metabolisms. Overall, our data suggest that STK4 signaling transiting through or initiating from a different subcellular compartment may result in distinct gene expression patterns and cellular biology.

PC is the most commonly diagnosed malignancy and the second leading cause of cancer death among men in the US [52]. Because androgen hormone signaling plays a critical role in PC development, progression, and metastasis, androgen deprivation is the first line of therapy for patients with locally advanced disease [45, 46]. However, almost all patients who receive primary androgen deprivation therapy (ADT) develop metastatic CRPC [45, 46]. Evidence indicates that AR is still a key driver of metastatic CRPC cell growth and survival, even in the absence of sub-physiological levels of androgens and in the presence of the second generation of ADT such as abiraterone acetate (ABI), a direct inhibitor of CYP17A that is a key enzyme in the androgen biosynthesis pathway [53, 54] and enzalutamide (ENZ), a direct AR inhibitor [55–57]. However, nearly all men with metastatic CRPC who are treated with ABI and ENZ also develop resistance to these agents, albeit with unknown mechanisms.

Here, we showed that enrichment of STK4 in the cytoplasm, lipid raft, and nucleus selectively regulates AR transcript. We also showed that STK4 signaling depending on its subcellular locations suppressed AR transcript with varying degrees. We noted that although CL-STK4 slightly increased AR transcript, LR-STK4 and NL-STK4 reduced it 4.6- and 7-fold, respectively, which correlated with the inhibition of AR target genes such as FKBP5, KLK3, and TMPRSS2 [43, 58]. However, many AR targets were minimally affected or unaltered by the targeted STK4 signaling (Table 3), suggesting that the regulation of AR targets by STK4 is selective or context-dependent. Previously, we reported that crosstalk between YAP1 and AR signaling could contribute to CRPC [29]. In that study, we showed that STK4 depletion increased YAP1/AR interaction, which coincided with CRPC cell growth. Therefore, it is possible that YAP1 may function as a key intermediate for the selective regulation of AR targets by STK4, but this warrants further investigation, which is not the subject of the current study.

Alterations of the DNA methylation, DNA repair, PI3K/AKT, RAS/RAF, WNT, and cell-cycle pathways are commonly observed both in primary prostate tumors [45] and metastatic CRPC [46]. Aberrant expression of the DNA repair genes such as MLH1 and MSH2 is implicated in advanced PC [59]. Here, we showed that LR-STK4 and NL-STK4, but not CL-STK4, reduced the expression of MLH1 about 5-fold while increasing MSH2 expression about 4-fold under the same growth conditions. Similarly, altered ZBTB16 (also known as PLZF1) signaling is also implicated in metastatic CRPC [46]. Induction of LR-STK4 and NL-STK4, but not of CL-STK4, downregulated the expression of ZBTB16 3.2-fold. ZBTB16 is an AR target gene [46]. Furthermore, CDKN2A and CDKN2B are potent suppressors of cell-cycle progression [46, 60]. LR-STK4 and NL-STK4 increased the expression of CDKN2B 3.4-fold. These findings suggest that STK4 restricts aggressive cancer cell growth by modulating key oncogenic pathways including DNA repair and cell cycle regulators.

**Fig 9:**
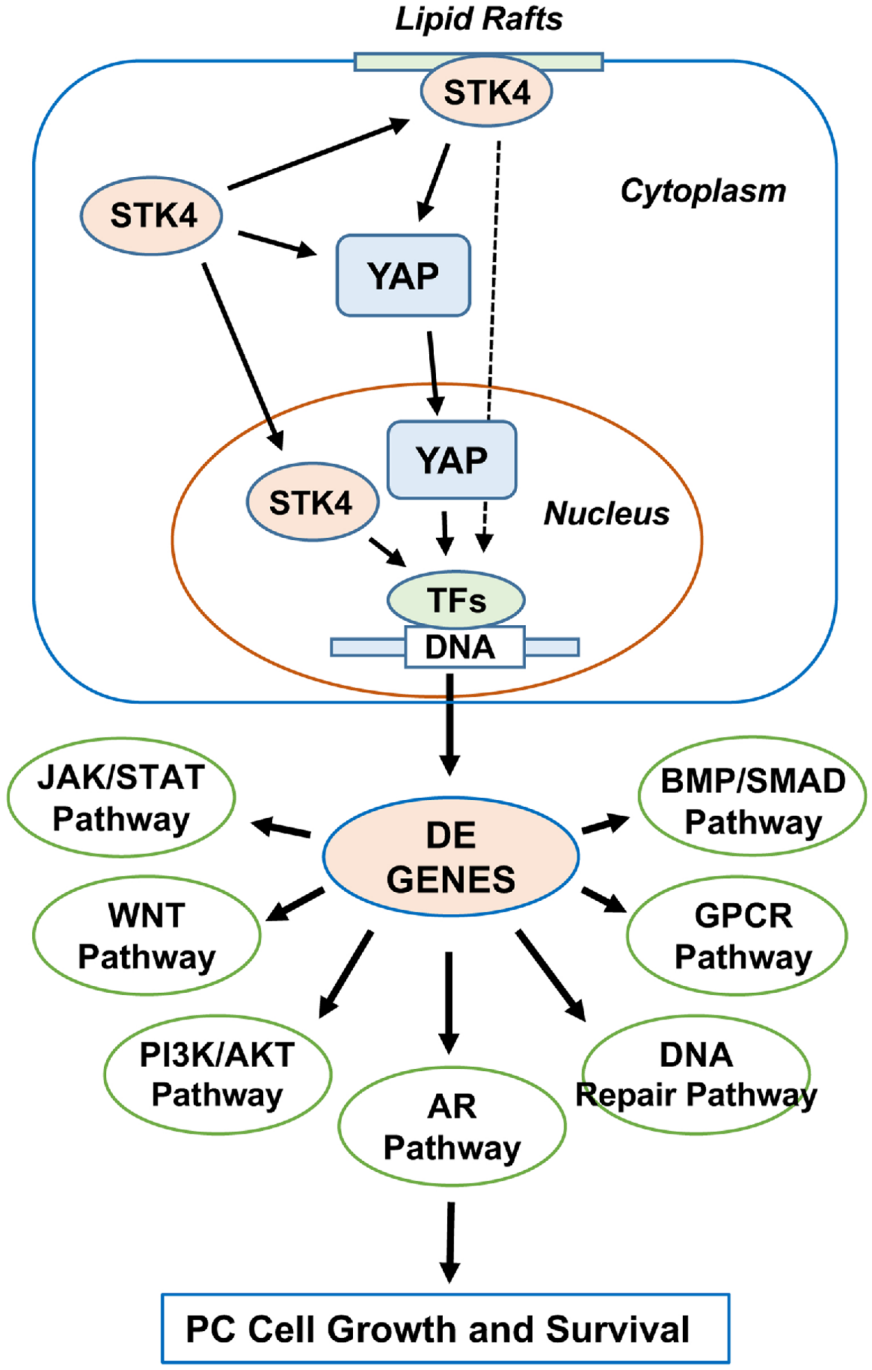
Schematic representation of the current study. TFs: Transcription factors. DE: Differentially expressed.

STK4 is a key negative regulator of YAP1/WWTR1-mediated gene transcription and oncogenesis. Herein, our data demonstrated that ectopic STK4 protein enriched in the cytoplasm, lipid raft, and nucleus had differential effects on YAP1/WWTR1 and YAP1/WWTR1-dependent gene expression. We noted that unlike NL-STK4, CL-STK4 and LR-STK4 showed similar trends in regulating YAP1 expression, although about 90% of genes regulated by LR-STK4 and NL-STK4 overlapped. These observations suggest the possibility that (a) CL-STK4 regulates gene expression by signaling through YAP1/WWTR1, (b) LR-STK4 regulates gene expression by YAP1/WWTR1-dependent and YAP1/WWTR1-independent mechanisms, and (c) NL-STK4 most likely regulates gene expression independently of YAP1/WWTR1. Nevertheless, future studies are necessary to test these hypotheses. In summary, we identified several DE genes and molecular pathways that are responded to the targeted STK4 expression and these pathways are known to be biologically and clinically relevant to human cancer including PC. The model in Fig 9 summarizes our main findings.

## Supporting information

S1 Fig Correlations of gene counts between technical replicates. Scatterplot of gene counts from the technical replicates show a high degree of correlation in all three conditions.
(TIF)

S2 Fig KEGG Hippo signaling pathway. Each gene is colored by gene expression fold change in LR-STK4 condition. Red: Upregulated, green: downregulated, white: not differentially expressed. Edge labels “e”: expression interaction, “+p”: phosphorylation, “-p”: dephosphorylation.
(TIF)

S1 Table Fold change and p-value results from RNAseq data in CL-STK4, LR-STK4 and NL-STK4 conditions. (XLSX)

S2 Table List of DE genes in CL-STK4. (XLSX)

S3 Table List of DE genes in LR-STK4. (XLSX)

S4 Table List of DE genes in NL-STK4. (XLSX)

S5 Table List of significantly enriched KEGG pathways in CL-STK4. (XLSX)

S6 Table List of significantly enriched KEGG pathways in LR-STK4. (XLSX)

S7 Table List of significantly enriched KEGG pathways in NL-STK4. (XLSX)

S8 Table List of significantly enriched GO terms in CL-STK4. (XLSX)

S9 Table List of significantly enriched GO terms in LR-STK4. (XLSX)

S10 Table List of significantly enriched GO terms in NL-STK4. (XLSX)

S11 Table List of YAP1/WWTR1 targets that are altered by CL-STK4, LR-STK4 and NL-STK4 expression in all three cell compartments. (XLSX)
