# Supplementary figures and images for: Mapping the STK4/Hippo signaling network in prostate cancer cell
Source: PLoS One. 2017 Sep 7;12(9):e0184590. doi: 10.1371/journal.pone.0184590 (PMC5589252; doi:10.1371/journal.pone.0184590)

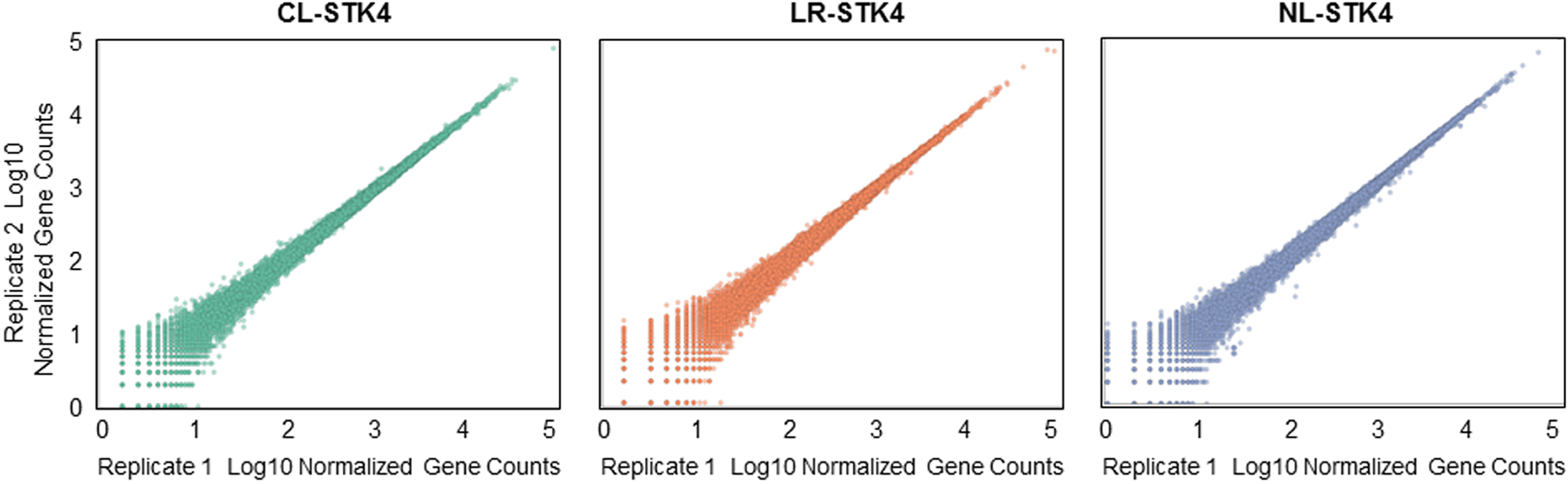

Supplement: S1 Fig — Scatterplot of gene counts from the technical replicates show a high degree of correlation in all three conditions. (TIF) [file pone.0184590.s001.tif]

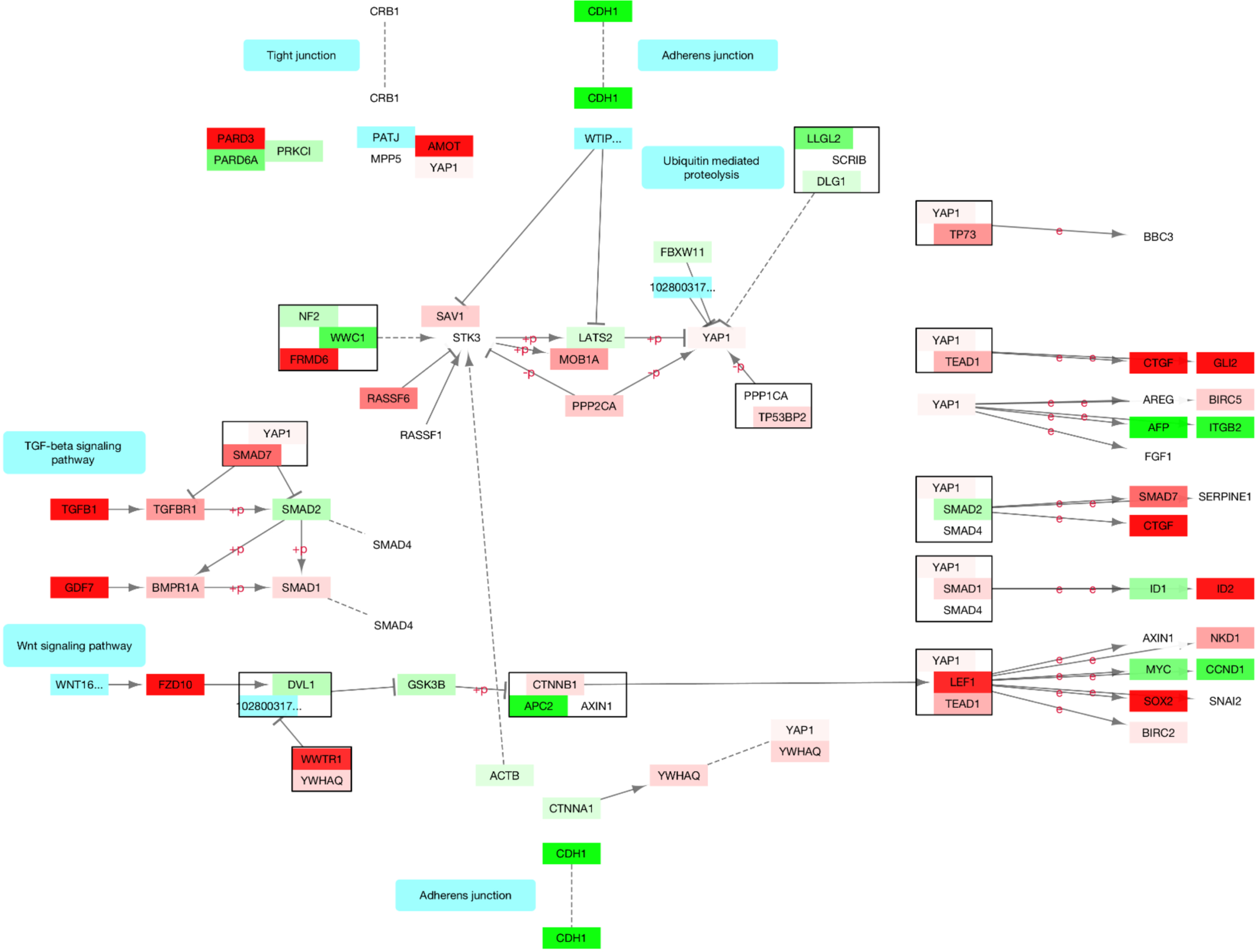

Supplement: S2 Fig — Each gene is colored by gene expression fold change in LR-STK4 condition. Red: Upregulated, green: downregulated, white: not differentially expressed. Edge labels “e”: expression interaction, “+p”: phosphorylation, “-p”: dephosphorylation. (TIF) [file pone.0184590.s002.tif]
